# Integration Strategy Is a Key Step in Network-Based Analysis and Dramatically Affects Network Topological Properties and Inferring Outcomes

**DOI:** 10.1155/2014/296349

**Published:** 2014-08-27

**Authors:** Nana Jin, Deng Wu, Yonghui Gong, Xiaoman Bi, Hong Jiang, Kongning Li, Qianghu Wang

**Affiliations:** ^1^Bioinformatics Department of School of Basic Medical Sciences, Nanjing Medical University, Nanjing 210029, China; ^2^College of Bioinformatics Science and Technology, Harbin Medical University, Harbin 150081, China

## Abstract

An increasing number of experiments have been designed to detect intracellular and intercellular molecular interactions. Based on these molecular interactions (especially protein interactions), molecular networks have been built for using in several typical applications, such as the discovery of new disease genes and the identification of drug targets and molecular complexes. Because the data are incomplete and a considerable number of false-positive interactions exist, protein interactions from different sources are commonly integrated in network analyses to build a stable molecular network. Although various types of integration strategies are being applied in current studies, the topological properties of the networks from these different integration strategies, especially typical applications based on these network integration strategies, have not been rigorously evaluated. In this paper, systematic analyses were performed to evaluate 11 frequently used methods using two types of integration strategies: empirical and machine learning methods. The topological properties of the networks of these different integration strategies were found to significantly differ. Moreover, these networks were found to dramatically affect the outcomes of typical applications, such as disease gene predictions, drug target detections, and molecular complex identifications. The analysis presented in this paper could provide an important basis for future network-based biological researches.

## 1. Introduction

Molecular interactions, such as protein-DNA interactions [1], protein-RNA interactions [2], DNA-DNA interactions [3], RNA-RNA interactions [4], and protein-protein interactions [5], facilitate various organismal functions, including the process of transcription [6], multiple long-range interactions between promoters and distal elements [7], and the regulation of gene expression [8]. Therefore, many experiments have been designed to detect intercellular and intracellular molecular interactions [9, 10]. Of these molecular interactions, protein-protein interactions have especially been found to play a crucial role in defining most of the molecular functions [11].

Consequently, molecular networks based on these interactions have been built to elucidate their underlying roles in biology [12]. Interactions have been utilised to build many protein-protein interaction network databases, such as DIP [13], HPRD [14], BIND [15], BioGRID [16], IntAct [17], and MINT [18], yielding more than 150,000 binary interactions. Researchers have used the interaction network of these databases to perform many studies and applications [11, 19, 20]. The interactions reported in these databases are derived from sources including yeast two-hybrid, anti-tag coimmunoprecipitation, mass-spectrometric, and literature mining experiments.

Traditional protein-protein interactions have been detected in a high-quality manner based on top-down- and hypothesis-based methods supported by experimental data [11]. Further, recent protein-protein interaction data have been generated in large numbers based on high-throughput methods, thus reconfiguring the biological network from a different point of view. However, two major shortages cannot be ignored. The coverage of current protein-protein interaction networks is less than 50%, and the accuracy of these data ranges from 10% to 50% [21–23]. Consider an example in which, by utilising various methodologies, researchers identify 80,000 protein interaction pairs in yeast; however, they only confirm interactions for approximately 2,400 pairs using more than two methods [22]. Several reasons can cause this situation. First, some data sources are not completely annotated. Second, each method has its own bias, meaning that each method can identify a subset of specific interactions. Third, large portions of the resulting dataset suffer from a high false-positive rate [22, 24, 25].

Comprehensive consideration of these data sources by the use of integration algorithms can solve the data bias inherently when using only a single data source and can also effectively increase the coverage of the interactome and decrease the false-positive rate [26]. Therefore, the development of new statistics and computational methods for integrating data from different databases is urgently needed and is a subject of concern in the present study [27]. Previous studies have directly utilised integration strategies that have not been properly evaluated, such as the intersection set of different networks (Intersection), the union set of different networks (Union), voting (which is a choice made by a network, Vote) [26], and the integration strategies based on Naive Bayes [28], Bayesian Networks [29], Logistic Regression [30], SVM [21], and decision trees, including Random Trees [31], Random Forest, and J48 [24]. For example, Lin and Chen applied a tree-augmented naive Bayesian (TAN) classifier to integrate heterogeneous data sources and generated fair results [28]; Wu et al. used SVM and Bayesian classifiers to detect whether a protein-protein interaction was reliable [21]; Gerstein et al. considered that voting did not take full advantage of the data source information in the process, and therefore, cannot generally obtain good results [32]; Ben-Hur and Noble deemed that SVM adopted different kernel functions depending on different integration tasks [33]; Jansen et al. and Rhodes et al. regarded that the premise of Naive Bayes was that the conditional probability of each attribute was independent [29, 34]; Sprinzak et al. thought that Logistic Regression actually was a generalised linear statistical model [30]; Chen and Liu believed that Random Forest combined many decision trees to enhance the correct rate of classification [35]; and by evaluating the precision, recall and area under the curve (AUC) scores of Support Vector Machine (SVM), Naive Bayes, Logistic Regression, Decision Tree, and Random Forest when predicting interactions, Qi et al. determined that Random Forest ranked as the top classifier for integration [24].

Although various types of integration strategies have been applied to the current research, the method of choice has not been considered. Although some researchers have simply evaluated some integration results, the comprehensive topological properties of the networks for different integration strategies and the impact of the outcomes of the typical applications based on these networks have not been rigorously evaluated.

In this paper, we combined 37 features representing 10 distinct groups of biological data sources based on former studies [24, 36, 37], including gene expression, physical interactions, domain interactions, HMS_PCI mass, TAP mass, yeast two-hybrid, genetic interactions, gene ontology (GO) annotations, and gene context analysis, to predict the more reliable protein-protein interactions. Our method utilised gold standard data sets and 11 commonly used methods (Union, Intersection, 2-Vote, 3-Vote, Naive Bayes, Bayesian Networks, Logistic Regression, SVM, Random Tree, Random Forest, and J48) from two types of integration strategies (empirical and machine learning) to integrate all of the interactions in previously mentioned databases; 2-Vote and 3-Vote indicate interactions that were supported by two and three databases, respectively. For seven machine-learning methods, we systematically evaluated the accuracy of correct classification, the area under the receiver operating characteristic (ROC) curve, the precision rate, recall rate, and the true-positive to false-positive ratio. To gain a more detailed understanding of the differences between these 11 new networks, we also compared the differences among their topological properties. For these integration strategies, topological properties, such as the number of proteins and interactions, the clustering coefficient, network density, average degree, and average path length, differed significantly between the different networks. Moreover, by analysing the ranks when predicting disease genes, searching for differences in detecting drug targets, and researching the modules for identifying molecular complexes, we found that the networks dramatically affected the outcomes of these typical applications. For example, when using phenotype similarity to detect disease genes, we obtained four different genes that were ranked as the top candidate in each of the 11 integration strategies. Compared to previous studies, the present study focuses more on the influence of different network integration strategies on typical biological applications, providing a novel perspective from which protein networks are studied from different viewpoints and an important basis for future network-based biological research.

## 2. Materials and Methods

### 2.1. DIP Database

DIP records experimentally detected protein interactions. Because the CORE set in the DIP database had been widely used to develop the prediction methods by the high-quality, high-throughput protein interaction data of it, and to study the properties of protein interaction networks, we selected the CORE set as the positive set for a gold standard database.

### 2.2. NEGATOME Database

The NEGATOME collects proteins that are experimentally supported noninteracting protein pairs via manual literature mining and analysing protein complexes from the RCSB Protein Data Bank (PDB). Because the manual dataset in the NEGATOME database does not contain high-throughput data and describes the unlikely direct physical interactions circumscribed only to mammalian proteins, most of which in this database are* Homo sapiens*, we selected the manual dataset as the negative set for a gold standard database.

### 2.3. Testing Datasets

We used five sources (HPRD, BIND, MINT, IntAct, and BioGRID) for protein-protein interaction data, representing most of the authoritative databases. These databases contain data derived almost from high-throughput experiments based on literature mining, yeast two-hybrid, mass spectrometric, and anti-tag coimmunoprecipitation experiments. However, approximately half of the interactions obtained from high-throughput experiments may represent false-positives as estimated by Von Mering et al. Therefore, it is critical to determine whether the interactions are authentic or pseudo.

### 2.4. Gene Expression

Genes that are mRNA coexpressed typically indicate protein interactions [38]. We collected 28 gene expression profiles, including more than 5000 samples of different tissues from the Gene Expression Omnibus (GEO) (http://www.ncbi.nlm.nih.gov/geo/), as previously described by Xu et al. [36]. Each gene containing a missing value was deleted, and all of the expression values were log-2 transformed. We combined any probes containing the same Gene Identifier. We then calculated the Pearson correlation coefficient (PCC) between each pair of genes to obtain a correlation coefficient matrix.

### 2.5. Domain-Domain Interactions

A domain is a structural or functional protein subunit, and the interaction between two proteins often involves binding between pairs of their constituent domains. Therefore, the selection of domains as characteristics is credible. We obtained domain information from the PFAM database, which is a large collection of protein families and is authoritative about domains. Additionally, domain-domain interaction information was obtained from the DOMINE database, which is a database of known and predicted protein domain (domain-domain) interactions. The database contains interactions inferred from PDB entries and those that were predicted by 13 different computational approaches using PFAM domain definitions. It contains 26,219 domain-domain interactions among 5,410 domains.

### 2.6. Physical Protein-Protein Interactions

We collected all of the interactions from the BioGRID, BIND, IntAct, HPRD, and MINT databases. All of the interactions that were not mapped to homologous human interaction proteins by HomoloGene (http://www.ncbi.nlm.nih.gov/homologene) in NCBI [39] were deleted. The physical protein-protein interaction scores ranged from 0 to 5; 0 meant that the interaction was from none of these databases, while 5 meant that the interaction was supported by each of these databases.

### 2.7. High-Throughput Direct PPI Dataset

The high-throughput direct PPI dataset contains two types: (1) derived from mass spectrometry and (2) derived from Y2H. In the mass spectrometry dataset, two subdatasets, TAP [40] and HMS-PCI [41], utilised two different protocols for this technique. We used the high-throughput PPI dataset provided by Qi et al. [24].

### 2.8. Human Phenotype

Function deletion in interactions or functionally related proteins frequently resulted in similar phenotypes [41–43]. We mapped the interactions that Han et al. attributed to homologous human interactions to obtain more accurate results.

### 2.9. Genetic Interactions

A synthetic genetic analysis (SGA) was used to reveal genetic interactions in* Saccharomyces cerevisiae *[44, 45]. Some reports have demonstrated a significant overlap between protein-protein interactions and genetic interactions [46]. Therefore, most neighbours of genetic interaction genes can be used to predict protein-protein interactions [45].

### 2.10. Biological Functional Annotation

Compared to interactions of different biological functions, protein-protein interactions are more likely to occur in proteins with similar biological functions. Moreover, proteins sharing a more specific annotation tend to interact with each other compared to those that share a more common annotation.

### 2.11. Gene Context Analysis

The gene context is based on genome sequences to infer* in silico* protein-protein interactions [22]. The gene context includes three types: gene fusion, gene cooccurrence, and gene neighbourhood.

Human phenotype, genetic interaction, biological functional annotation, and gene context analyses have been previously performed by Xia et al. [37] to predict interactions from model organisms.

To avoid the impact of human factors on the results analysis, we constructed all the machine learning integration strategies with a unified software platform, WEKA (Waikato Environment for Knowledge Analysis), which is widely used in classification. WEKA, a public data-mining platform, collects a large number of machine learning algorithms that are used to undertake the task of data mining, including preprocessing, classifying, clustering, associating, attribute selecting, and visualising.

### 2.12. Seven Machine Learning Classifiers Constructed by Using the Gold Standard Datasets

**Figure 1:**
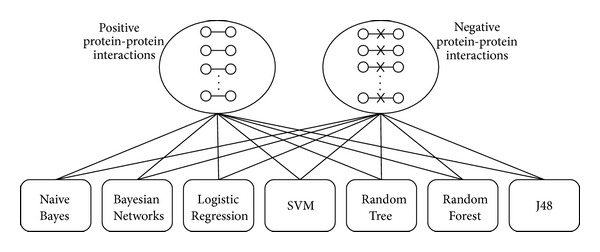
Seven machine learning classifiers constructed by using the gold standard datasets. The gold standard datasets, positive protein-protein interactions in the DIP database, and negative protein-protein interactions in the NEGATOME database were used to construct the seven machine learning classifiers based on the following methods: Naive Bayes, Bayesian Networks, Logistic Regression, Support Vector Machine (SVM), Random Tree, Random Forest, and J48.

All of the protein data that we obtained were derived from different databases, and each database has its own presentation pattern, such as Gene Identifier, Gene Symbol, Accession Number, and UniProtKB Number. To unify the data, we converted all of the protein presentation patterns into Gene Identifiers. Then, we deleted any interactions that were not mapped to the Gene Identifiers or homologous human interactions by NCBI HomoloGene. After obtaining the gold standard protein networks, we constructed seven different machine learning classifiers using seven integration strategies (Naive Bayes, Bayesian Networks, Logistic Regression, SVM, Random Tree, Random Forest, and J48) (Figure 1).

## 3. Results

In this study, we used four empirical integration strategies and seven machine learning classifiers constructed by the reliable positive and negative gold standard sets from DIP and NEGATOME, respectively, to integrate the protein-protein interaction networks. Some indicators, such as the accuracy of those correctly classified, the area under the ROC curve, and the precision and recall rates, are typically used to evaluate a supervised machine learning method; therefore, we initially evaluated the performance of these seven machine-learning classifiers in these ways.

### 3.1. Performance of the Classifiers Constructed by Seven Machine Learning Integration Strategies

**Figure 2:**
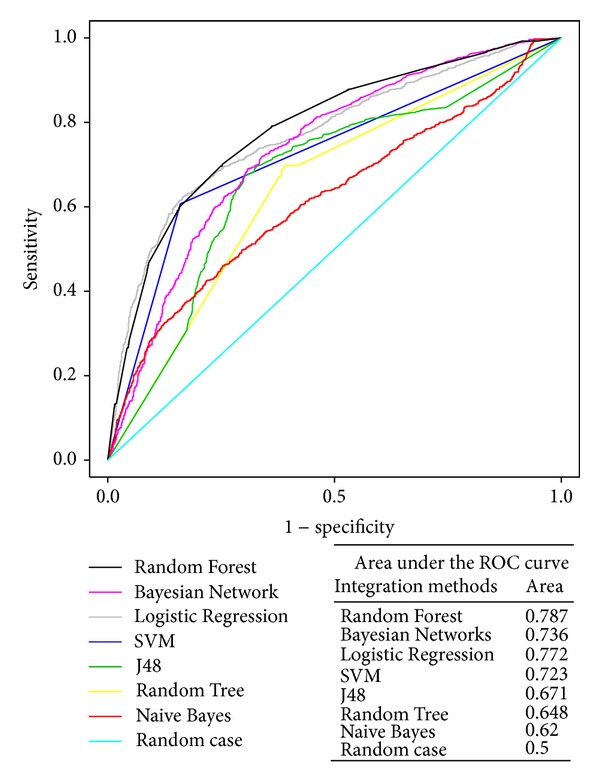
ROC curves for seven machine learning integration strategies using 10-fold cross-validation against the gold standard datasets. Each point on the ROC curves of the seven integration strategies is created by the unique sensitivity and specificity against a specific likelihood ratio cut-off. Each name of the curve derived from the different integration strategies is shown in the legend. The different colours stand for the different curves for the different strategies. The area under the curve is also presented in the figure. Sensitivity and specificity are calculated during the 10-fold cross-validations.

From the seven different integration strategies, the seven classifiers showed quite different classification results. The ACC score, AUC score, precision and recall rates, and TP/FP score of each integration strategy were significantly distinct (Figure 2, Table 1). For example, the ACC score ranged from 0.5391 in Naive Bayes to 0.7196 in Random Forest, and the area under the ROC curve ranged from 0.62 in Naive Bayes to 0.787 in Random Forest. Therefore, different integration strategies affect the outcome of the classification.

### 3.2. Eleven New Networks Built by Empirical and Machine Learning Integration Strategies

**Figure 3:**
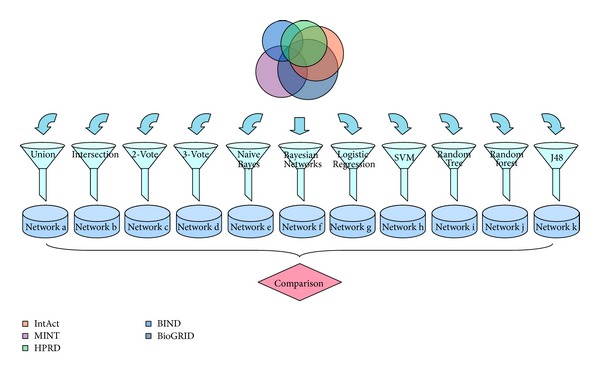
Eleven new networks built by empirical and machine learning integration strategies. Eleven new networks constructed by funnel-like empirical and machine learning integration strategies, namely, Union, Intersection, 2-Vote, 3-Vote, Naive Bayes, Bayesian Networks, Logistic Regression, SVM, Random Tree, Random Forest, and J48, from the entire set of data in the IntAct, MINT, HPRD, BIND, and BioGRID databases.

After inputting 145,534 interaction pairs from the five databases into these funnel-like classifiers, we obtained 11 different new networks (Figure 3). As shown in Table 2, the 11 new networks are significantly different from each other. Although the input network was constant, the ratio of the number of predicted protein pairs to the originally considered protein pairs was remarkably discrepant. It is clear that the differences between the seven machine learning networks are not significant; in other words, the machine learning strategies are somewhat stable. For example, the coverage of each network range from 0.7874 (Random Tree) to 0.9773 (SVM); however, most of the coverage in the machine learning strategies were approximately 95%. However, a remarkable distinction was present in four empirical strategies. For example, Intersection considered only 0.34% of the interactions to be true interactions, 2-Vote considered 28.01% of the interactions to be true interactions, and Union considered all of the interactions to be true interactions.

Seven machine learning networks in Table 3 show that although a certain ratio of repeats was observed, the interactions in each machine learning network were not the same. For example, compared with the original network, the number of interactions that did not appear in any of the machine learning classifier outputs was 1,808 (1.24% of the total), while the number of interactions in all of the classifiers' output was 89,740 (61.66% of the total). As indicated in all 11 integration networks in Table 3, in all of the networks, including empirical and machine learning strategies, the number of interactions varies among 11 networks generated by different integration strategies. For example, the number of interactions that appeared in eight networks was 71,478 (49.11%), and the number of interactions that appeared in 11 networks was 312 (only 0.22%).

### 3.3. Topological Properties of the 11 Empirical and Machine Learning Networks

We first analysed the network topological properties of each integration network; the two most critical attributes in the network are the distance and the number of connections [47]. Almost all of the other topological properties are based on these two properties. We calculated the number of proteins and interactions, network diameter, average degree, network density, average path length, and global clustering coefficient for each network (Table 4).

The network diameter is the maximum eccentricity of any point in the protein network. It represents the greatest distance between protein pairs. The density of a protein network is the total number of interactions divided by the total number of possible interactions. The average path length represents the average distance of the shortest path between all of the node pairs. Additionally, it provides the overall efficiency of information or mass transport in a network. The global clustering coefficient represents the degree to which the proteins in a protein network tend to cluster together.

Tremendous differences were found between the 11 networks of empirical and machine learning strategies. For example, the number of proteins in the networks integrated by machine learning strategies ranged from 507 to more than 14,000, and the average degree ranged from 1.96 to more than 15. Additionally, the average path length and clustering coefficient were dramatically varied.

If we account only for the networks that were integrated by the empirical strategies, almost all of the properties were dramatically varied; for example, the range of changes in the number of proteins and the interactions were especially large. Dramatic variation was also observed in the remaining properties, such as the network diameter, average degree, network density, average path length, and clustering coefficient.

Considering only the networks integrated by the machine learning strategies, although some properties, such as the number of proteins, the network diameter, the network density and the shortest path length, did not vary, other properties varied dramatically. For example, the number of interactions ranged from 114,598 in the Random Tree to 142,226 in the SVM network; the average degree of the networks ranged from 15.82 in the Random Tree network to 19.10 in the SVM network; and the clustering coefficient ranged from 0.0156 in the Random Tree network to 0.0204 in the Bayesian Networks network and the SVM network.

Therefore, the alteration in the topological properties reveals that the different integration strategies dramatically affect the outcomes. These integration strategies affect the number of proteins and interactions in the networks, consequently affecting the network aggregation, mass transport, and connectivity.

### 3.4. Detection of Disease Genes Using Phenotype Similarity Based on Networks

Protein interaction data are ultimately integrated to facilitate actual applications. The advancement of biotechnology enables the proteome scope of protein interaction networks, making the networks become more and more attractive to researchers studying systems biology [48]. Typically, researchers tend to use protein interaction networks to identify disease candidate genes [49, 50], drug targets [51, 52], and functional modules [53].

The prediction of disease genes based on a protein network is an important typical application of biological networks [54] and is also vital to the development of physianthropy [20]. To detect disease genes, we utilised the method of Lage et al. [54], which is based on phenotype similarity. In this method, disease gene prediction is accomplished based on the assumption that proteins that are directly connected to disease proteins tend to have the same disease phenotype as the disease protein [55–58].

We used epithelial ovarian cancer (EOC) as a specific case. After downloading all 301 genes in the linkage interval on 3p25-22 [59] from GENE of NCBI [60], we identified the subnetworks of these 301 genes in the 11 networks obtained from the 11 integration strategies. Then, a score was obtained for every interaction that was an edge in the subnetworks via Kasper's scoring rules. Next, according to the method described by van Driel et al. [61], which is based on OMIM [62] and MeSH [63], we calculated the similarity between each phenotype and EOC as the score of the protein that was the node in the subnetworks [64]. According to the following formula, we obtained the final score for each candidate gene:

(1) $$\begin{array}{c} \text{Score}=\overset{N}{\underset{i=1}{\sum }}S_{i}P_{i}, \end{array}$$

where *N* is the number of partners connected to the candidate gene, *S*_*i*_ is the interaction score, and *P*_*i*_ is the protein score.

Finally, we sorted the entire candidate genes to identify the one that had the highest score.  Table 5 lists the highest scoring candidate genes for each integration network.

These findings clearly revealed that four different genes, ATP2B2, MYD88, TGFBR2, and GRM7, were ranked as the top genes in each of 11 integration strategies. This finding indicates that the empirical and machine learning strategies dramatically affected the overall outcomes. Separately, seven machine learning strategies mainly identified ATP2B2 as the top gene; however, Random Tree identified GRM7 as the top gene; overall, this result was relatively stable. However, four empirical strategies yielded three different top genes, indicating that the empirical strategy was quite unstable and seriously impacted the reliability of the results.

### 3.5. Detection of Disease Genes Using a Network-Based Random Walk with Restart (RWR)

Based on an early disease-gene screening method based on phenotype similarity or network topological properties and the advances of genome sequencing, gene expression analysis and other parallel technologies, it is clear that new disease-gene screening methods are emerging [54, 65, 66]. Well-known studies have demonstrated that the RWR method is superior to other methods, such as methods based on clustering or based on neighbouring nodes [66]. Therefore, we used the RWR method to screen for causative disease genes.

RWR refers to a process in which a given node in a network is used as a starting point upon which iterations are performed; at each iteration, the current node is used as a starting point for a transfer to a randomly selected adjacent node as follows:

(2) $$\begin{array}{c} p^{t+1}=\left(1-r\right)Wp^{t}+rp^{0}, \end{array}$$

where *p* ^*t*^ is a vector that represents the probability of a certain node being the random walk node at time *t* in the network, *r* represents the probability of the random walk node returning to the starting node at any moment, and *W* represents the adjacency matrix after the column standardisation of the network.

**Figure 4:**
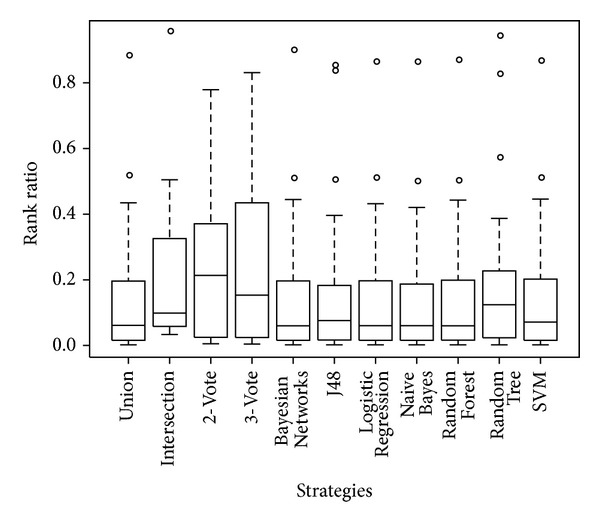
The performance of detecting disease gene using RWR. We used a box-plot to show the rank difference between each of the 11 integration strategies. Apparent distinctions exist between the different networks by different integration strategies.

We selected 29 disease genes of brain tumours, including neurofibroma, glioma, glioblastoma, and astrocytoma, from the Cancer Gene Census (CGC) [67, 68]. One gene was randomly selected to verify the prediction efficiency, and the remaining genes were used as seeds for the RWR algorithm. The number of repetitions depended on the number of remaining genes. We considered whether the integration strategy was outstanding based on the ratio of the average position of a nonseed gene in these repeated experiments to the total number of nodes (Table 6, Figure 4).

By comparing the rank ratios in Table 6 and Figure 4, it is clear that (A) the average rank of the remaining (single) disease gene was approximately 14% of the total number of nodes, indicating a satisfactory performance at discovering disease genes by the RWR method, and (B) although some of the machine learning strategies were stable in the rank ratio (e.g., the rank ratio of Naïve Bayes, Logistic Regression, SVM, Random Forest, and Bayesian Networks were approximately 0.14), the rank ratios of the other two machine learning strategies were 0.1627 in J48 and 0.1862 in Random Tree; these values were significantly different from the former five strategies. Furthermore, the rank ratios of four empirical strategies were remarkably distinct from the machine learning strategies; for example, the ratio of 3-Vote, 2-Vote and Intersection were approximately 0.24. Therefore, the different strategies greatly impacted the rank of the nonseed gene by the RWR method.

We next selected all of the disease genes as seeds to obtain the top 10 genes for all of the generated networks except for the seeds (Table 7). Table 7 indicates that the gene lists discovered by the empirical methods are significantly different from the gene lists discovered by the machine learning methods. For example, the genes identified by empirical strategies, such as YWHAB, RAD50, YWHAZ, YWHAE, ERBB2, and RB1, were not identified by any of the machine learning strategies. The top 10 genes detected by the four empirical strategies were also remarkably distinct; for example, UBC, TAF1, MYC, and HNF4A were identified by Union but were not identified by any of the other empirical strategies. Although the genes that were identified by the machine learning methods shared some overlap, different methods also identified different genes; for example, DTNBP1 was only identified by J48. Therefore, the different strategies dramatically impacted the top 10 genes identified by the RWR method.

### 3.6. The Approach of Discovering Drug Targets Based on Network Topology Properties

As mentioned above, the identification of drug targets is one typical use of a protein interaction network. Similar to Zhu et al. [69], we applied the original protein network topology-based approach to identify drug targets.

Previous studies have shown that compared to general network proteins, drug target proteins are significantly different with respect to their topological properties. For example, the degree of a drug target is larger [70], the average distance and the shortest length between two drug targets are shorter than between a drug target and a general protein, the proportion of the target proteins in the neighbouring nodes of a target protein is significantly higher than the proportion of the target proteins in the neighbouring nodes of a general protein, and the clustering coefficient of a drug target is significantly lower than for a general protein [69].

We obtained the drug target information from DrugBank [71] on March 16, 2013; we then mapped these target genes to each protein interaction network. We next selected five measures to identify drug target proteins; these measures included the degree, 1N index, clustering coefficient, and the average distance and shortest path length between a protein and a drug target protein. The 1N index was the proportion of target proteins in the neighbouring nodes of a protein. We ranked each protein based on these five measures in each of the networks. We then determined the number of drug targets that were included in the top 100 proteins of each network (1N) and the proportion of target proteins in all of the proteins (Table 8).

Due to the characteristics of the drug targets, based on the machine learning strategies, the proportion of drug target proteins in the top-ranked 100 proteins was approximately 83%, and the target ratio was approximately 0.13. Additionally, there was only a small change between the different machine learning strategies; for example, Random Tree and J48 each identified 81 targets, while Logistic Regression identified 85 targets. However, the results obtained from the different empirical integration strategies were significantly different. For example, Union identified 82 drug targets in the top 100 proteins from a network containing 1,984 drug targets, while Intersection identified 40 drug targets in the top 100 proteins from a network containing 133 drug targets.

As shown in Table 9, the numbers of targets were different. For example, 58 drug targets were uniquely detected by one integration strategy, accounting for 33.91% of the 171 targets that appear in the top 100 of each network; however, only four drug targets were simultaneously detected by four integration strategies, accounting for 2.34% of the 171 targets that appear in the top 100 of each network. Therefore, the outcomes of the drug target discovery process were dramatically affected by the different strategies.

### 3.7. Identification of Molecular Complexes Based on the MCODE Clustering Algorithm

Molecular complexes are key elements in molecular function. Human disease is closely correlated with human molecular complexes, and molecular complexes are widely applied in molecular functional annotation and disease prediction. Therefore, it is critical to identify molecular complexes [72]. Because the protein interaction network contains functional annotation data, it is important to identify molecular complexes from protein interaction networks. Because a subunit of the protein exercises a biological function, the prediction of the function of unknown proteins has been demonstrated to be of great significance [73].

The identification of molecular complexes is an important application in biological networks. For example, Wu et al. compiled the redundant human complexes to build a comprehensive catalogue and then investigated the relationship between protein complexes and drug-related systems [72]. Song and Singh analysed proteins, complexes, and processes and considered physical interactions within and across complexes and biological processes to understand the protein essentiality [74]. Zhang and Shen analysed functional modules based on a protein-protein network analysis in ankylosing spondylitis [75].

In this paper, we utilised a Cytoscape [76] plug-in called MCODE, which is based on the MCODE [73] clustering algorithm; this plug-in mines tightly connected regions in protein interaction networks that represent molecular complexes. Cytoscape is free software program that graphically displays, edits, and analyses networks. It supports a variety of network description formats, and the user can add rich annotation information to the networks. a large number of functional plug-ins that were developed by developers and third parties can be used for in-depth analysis of network problems. We analysed the topological properties of each single top molecular complex in each network and compared their intersections (Table 10).

Table 10 reveals that the different networks obtained from different integration strategies affected the finding on the effect of molecular complexes. Overall, even though the diameters of the networks and the clustering coefficient were nearly identical, the number of proteins and interactions differed greatly in both the empirical and machine learning strategies. For example, only five proteins and nine interactions were identified in the molecular complexes mined by Intersection, and only 40 proteins and 702 interactions were identified by Random Tree; however, Union identified 63 proteins and 1,721 interactions, and Bayesian Networks identified 65 proteins and 1,787 interactions when identifying molecular complexes. Additionally, the average degree of each network ranged from 3.6 in the Intersection network to 54.127 in the Union network and from 35.1 in the Random Tree network to 54.985 in the Bayesian Network. The network density and average path length also varied in both the empirical and machine learning strategies.

Table 11 lists the proteins that displayed the largest degree in each molecular complex of each network. It is clear that every molecular complex is different from the others because the proteins with the largest degree are different (Table 11).

The proteins displaying the smallest degree (4) were IRAK1, IRAK2, and IRAK3 (based on Intersection) in the molecular complex identification, while the proteins with the largest degree (64) were RPL5 and UBC by Union in molecular complex finding by empirical strategies. Additionally, Logistic Regression revealed that RPL5 had the largest degree (65), while Naive Bayes revealed that MED26 and MED29 displayed the smallest degree (27) by machine learning strategies. Therefore, large distinctions exist between the empirical and machine learning strategies when identifying molecular complexes.

## 4. Discussion

Protein-protein interaction studies act as new method for improving our understanding of molecular physiological processes. With the growing number of in-depth studies on protein-protein interaction networks, scientists are gaining knowledge of the interactions from various methods. Therefore, the key to network analyses is determining which integration strategy should be implemented. In this study, we analysed and evaluated the networks integrated by 11 commonly used strategies of two types of integration strategies, empirical and machine learning, including Union, Intersection, 2-Vote, 3-Vote, Bayesian Network, Support Vector Machine, Naive Bayes, Random Tree, J48, Logistic Regression, and Random Forest. By comparing the scores and the ranks, these strategies detected disease genes based on phenotype similarity and the RWR algorithm. Based on rank, the networks identified drug targets based on five measures, including average degree, 1N index, clustering coefficient, average path length, and shortest path; the topological properties of the molecular complexes that were identified were based on a Cytoscape plug-in called MCODE. Thus, we conclude that different integration strategies can obtain extremely different outcomes for these typical applications.

Most of the methods of the existing studies are to evaluate the character of the network itself. For example, Qi et al. found that Random Forest performed best of the six methods that they analysed [24]. Although Random Forest performed better based on ACC and AUC, with scores of 0.7196 and 0.787, respectively, subsequent evaluations confirmed that it is insufficient to determine the best integration strategy based solely on accuracy. Ultimately, one must also consider the comprehensive applications. Nevertheless, we did not only analyse the quality of the networks based simply on the integration of a wide range of data. In other words, although we analysed the AUC, accuracy, and topological properties, we also focused on typical practical applications, such as disease gene discovery, drug target detection, and molecular complex identification. We then compared the differences between the various networks in these applications. Therefore, this study is more biologically significant than previous studies, and it provides a novel perspective from which scholars can study protein networks.

It should be emphasised that a substantial amount of in-depth exploration of this topic remains. First, the integration strategies can be combined with other methods for further improvement. For example, the Naive Bayesian method used by Lin and Chen [28] is a tree-like Naive Bayesian method. Alternatively, a variety of integration strategies may be combined in a manner that emphasises the advantages of each integration strategy to improve the results of the integration. Second, because some features, such as phenotype similarity, genetic interaction, and shared GO annotation, which were utilised in IntNetDB described by Xia et al. [37], and TAP, HMS-PCI, and Y2H, which were utilised by Qi et al. [24], do not consider current data, deviations may exist in the results. However, our results are reliable because the same input data were used for all of the integration strategies; therefore, these deviations were not significant.

Although the processes of disease gene discovery and drug target detection revealed the stability of the seven machine learning strategies, these supervised machine learning strategies should have been similar; any difference between them warrants further examination. However, some properties used to identify molecular complexes have revealed the instability of several machine learning strategies. Almost all of the typical applications indicate that empirical strategies are quite unstable; however, these empirical strategies are applied in a substantial number of studies. Consequently, if these strategies are not evaluated, the resulting data will be unreliable, strongly influencing the studies.

Integration strategies are the key step in the network analysis, and they severely affect the outcomes of the various applications. Therefore, because technological advancement dictates the subsequent update of data and the integration strategies, the integration of the updated data becomes even more important. Software and websites that can rapidly integrate these updated data should be developed so that researchers can gain access to more reliable data and complete protein-protein interaction networks.

## Supplementary Material

OMIM descriptions of the top 10 disease genes that were detected by RWR on eleven integrated networks, seed genes for RWR algorithms were excluded.

## Figures and Tables

**Table 1:** Performance of the classifiers constructed by seven machine learning integration strategies.

Strategy	ACC	AUC	Precision	Recall	FP rate	TP/FP
Naive Bayes	0.5391	0.62	0.524	0.539	0.518	1.041
Bayesian Networks	0.6325	0.736	0.683	0.632	0.418	1.512
Logistic Regression	0.7188	0.772	0.724	0.719	0.275	2.615
SVM	0.7144	0.723	**0.738**	0.714	**0.267**	**2.674**
Random Tree	0.6568	0.648	0.656	0.657	0.35	1.877
Random Forest	**0.7196**	**0.787**	0.72	**0.72**	0.292	2.466
J48	0.6808	0.671	0.681	0.681	0.323	2.108

Note: ACC stands for the accuracy of the correctly classified items (after a 10-fold cross-validation). AUC indicates the area under the ROC curve. Precision is the number of true positives divided by the total number of elements labelled as belonging to the positive class. Recall (also referred to as the True Positive Rate) represents the number of true positives divided by the total number of elements that actually belong to the positive class. The FP rate indicates the false positive rate. TP/FP reveals the true positive to the false positive ratio. Bold type indicates the maximum value in the ACC, AUC, Precision, Recall, and TP/FP columns and indicates the minimum value in the FP rate column.

**Table 2:** The coverage of each network built by 11 integration strategies.

Strategy	Number	Coverage
Union	145534	1
Intersection	497	0.0034
2-Vote	40766	0.2801
3-Vote	12891	0.0886
Naive Bayes	134095	0.9214
Bayesian Networks	140956	0.9685
Logistic Regression	140746	0.9671
SVM	142226	0.9773
Random Tree	114598	0.7874
Random Forest	139082	0.9557
J48	120541	0.8283

Note: Number stands for the number of the predicted interaction pairs by each integration strategy. Percentage represents the ratio of the number of the predicted interaction pairs to the number of total interaction pairs in the five databases.

**Table 3:** The duplication of seven machine learning networks and all 11 integration networks.

Seven machine learning networks			All 11 integration networks		
DT	Number	Percentage	DT	Number	Percentage
0	1808	1.24%	1	1683	1.16%
1	1277	0.88%	2	134	0.09%
2	79	0.05%	3	718	0.49%
3	299	0.21%	4	777	0.53%
4	1410	0.97%	5	1234	0.85%
5	9434	6.48%	6	7653	5.26%
6	41487	28.51%	7	32465	22.31%
7	89740	61.66%	8	71478	49.11%
			9	20680	14.21%
			10	8400	5.77%
			11	312	0.22%

Note: DT stands for the number of times in which all of the interactions were duplicated. Number represents the number of such interactions. Percentage reveals the ratio of the number of such interactions to the total number in the original network.

**Table 4:** The topological properties of the 11 new empirical and machine learning networks.

	Empirical				Machine learning						
	Union	Intersection	2-Vote	3-Vote	Naive Bayes	Bayesian Networks	Logistic Regression	SVM	Random Tree	Random Forest	J48
Proteins	**14936**	507	9548	5558	14840	14869	14890	**14895**	14486	14860	14570
Interactions	**145534**	497	40766	12891	134095	140956	140746	**142226**	114598	139082	120541
Diameter	15	6	**16**	15	15	15	**16**	**16**	**16**	15	**16**
Degree	**19.49**	1.96	8.54	4.64	18.07	18.96	18.90	**19.10**	15.82	18.72	16.55
Density	0.00130	**0.00387**	0.00089	0.00083	0.00122	**0.00128**	0.00127	**0.00128**	0.00109	0.00126	0.00114
ASP	2.9216	**1.9164**	4.4487	4.7394	2.9372	2.9245	2.9256	**2.9217**	3.0447	2.9280	3.0103
CC	0.0206	**0.1262**	0.0471	0.0340	0.0161	**0.0204**	0.0197	**0.0204**	0.0156	0.0194	0.0170

Note: Proteins, Interactions, Diameter, Degree, and Density indicate the number of proteins, the number of interactions, the network diameter, the average degree and the network density, respectively. ASP and CC are the average path length and clustering coefficient, respectively. Bold type indicates the minimum value for average path length and the maximum value for the other topological properties of the empirical and machine learning methods.

**Table 5:** Description of the top genes in 11 integration networks from the detection of disease genes based on a phenotype similarity study.

Strategy	Gene symbol	Official full name
Union	ATP2B2	ATPase, Ca++ transporting, plasma membrane 2
Intersection	MYD88	Myeloid differentiation primary response 88
2-Vote	TGFBR2	Transforming growth factor, beta receptor II (70/80 kDa)
3-Vote	TGFBR2	Transforming growth factor, beta receptor II (70/80 kDa)
SVM	ATP2B2	ATPase, Ca++ transporting, plasma membrane 2
Naive Bayes	ATP2B2	ATPase, Ca++ transporting, plasma membrane 2
Random Tree	GRM7	Glutamate receptor, metabotropic 7
J48	ATP2B2	ATPase, Ca++ transporting, plasma membrane 2
Logistic Regression	ATP2B2	ATPase, Ca++ transporting, plasma membrane 2
Random Forest	ATP2B2	ATPase, Ca++ transporting, plasma membrane 2
Bayesian Networks	ATP2B2	ATPase, Ca++ transporting, plasma membrane 2

**Table 6:** The performance of the detection of disease genes using RWR.

Strategy	Rank	Nodes	Rank ratio
Naive Bayes	2065.86	14840	0.1392
Logistic Regression	2113.31	14890	0.1419
SVM	2133.86	14895	0.1433
Union	2146.21	14936	0.1437
Random Forest	2136.90	14860	0.1438
Bayesian Networks	2139.83	14869	0.1439
J48	2370.10	14570	0.1627
Random Tree	2697.93	14486	0.1862
3-Vote	1306.61	5558	0.2351
2-Vote	2245.89	9548	0.2352
Intersection	123.5	507	0.2436

Note: Rank indicates the average rank of the nonseed genes in several repeated experiments; the number of repetitions depended on the number of remaining genes. The rank ratio reveals the average rank divided by the total number of nodes in each network. The rank ratio was used to evaluate whether the performance of the integration strategy was outstanding. The smaller the scale is, the better the integration strategy is.

**Table 7:** The top 10 genes of all of the genes, except for the seed genes, from 11 integration networks in the detection of disease genes using RWR.

Strategy	The symbols of the top 10 genes
Union	UBC, TAF1, MYC, HNF4A, SMARCA4, ELAVL1, CDK2, FASLG, XRCC6, and SDHA

Intersection	YWHAB, RAD50, CTNNB1, GRB2, SHC1, ABL1, YWHAZ, YWHAE, ERBB2, and RB1

2-Vote	MLH1, PTPN6, XRCC6, EXO1, ARHGDIA, VAV3, HRAS, FASLG, APP, and TNIK

3-Vote	PTPN6, MAX, ZHX1, CCDC90B, MLH1, EXO1, IMMT, VIM, ASF1B, and ASF1A

SVM	UBC, TAF1, MYC, HNF4A, SMARCA4, ELAVL1, CDK2, FASLG, XRCC6, and SDHA

Naive Bayes	UBC, TAF1, MYC, HNF4A, SMARCA4, ELAVL1, CDK2, XRCC6, FASLG, and SDHA

Random Tree	UBC, MYC, XRCC6, SMARCA4, ARHGDIA, TAF1, ABL1, ELAVL1, FASLG, and CDK2

J48	UBC, TAF1, MYC, HNF4A, SMARCA4, XRCC6, ELAVL1, FASLG, CDK2, and DTNBP1

Logistic Regression	UBC, TAF1, MYC, HNF4A, SMARCA4, XRCC6, ELAVL1, CDK2, FASLG, ARHGDIA

Random Forest	UBC, TAF1, MYC, HNF4A, SMARCA4, ELAVL1, CDK2, FASLG, XRCC6, and SDHA

Bayesian Networks	UBC, TAF1, MYC, HNF4A, SMARCA4, ELAVL1, CDK2, FASLG, XRCC6, and SDHA

Note: the description of these genes was listed in Supplementary Table S1 of the Supplementary Material available online at http://dx.doi.org/10.1155/2014/296349.

**Table 8:** Performance of each network built by integration strategies for the discovery of drug targets based on topological properties.

Strategy	1N	Target	Node	Target ratio
Union	82	**1984**	**14936**	0.132833
Intersection	40	133	507	**0.262327**
2-Vote	79	1464	9548	0.153331
3-Vote	55	885	5558	0.15923
SVM	83	1974	14895	0.132528
Naive Bayes	83	1969	14840	0.132682
Random Tree	81	1941	14486	0.133991
J48	81	1945	14570	0.133493
Logistic Regression	**85**	1981	14890	0.133042
Random Forest	83	1972	14860	0.132705
Bayesian Networks	83	1973	14869	0.132692

Note: 1N indicates the number of targets included in the top 100 proteins. Target indicates the number of targets in the network. The target ratio reveals the percentage of targets in a network. The bold type indicates the maximum values in the 1N, Target, Node, and Target ratio columns.

**Table 9:** The duplication of targets in the top 100 in each network built by all 11 integration strategies.

DT	Number	Percentage
1	58	0.3391
2	21	0.1228
3	5	0.0292
4	4	0.0234
5	6	0.0351
6	3	0.0175
7	6	0.0351
8	27	0.1579
9	21	0.1228
10	11	0.0643
11	9	0.0526

Note: DT indicates the duplication times of the targets that appear in the top 100 of each network. Number represents the number of targets. Percentage reveals the ratio of the number of targets to the total of all of the targets that appear in the top 100 of each network.

**Table 10:** The topology properties of the molecular complexes found by 11 networks built by integration strategies based on the MCODE clustering algorithm.

	Empirical				Machine learning						
	Union	Intersection	2-Vote	3-Vote	Naive Bayes	Bayesian Networks	Logistic Regression	SVM	Random Tree	Random Forest	J48
Proteins	**63**	5	26	29	55	**65**	64	61	40	59	48
Interactions	**1721**	9	169	92	438	**1787**	1761	1628	702	1497	1028
Diameter	2	2	2	**7**	**5**	2	2	2	2	2	2
Degree	**54.127**	3.6	12.769	5.241	15.927	**54.985**	54.844	53.377	35.1	50.746	42.833
Density	**0.873**	0.9	0.511	0.187	0.295	0.859	0.871	0.890	0.9	0.875	**0.911**
ASP	1.127	**1.1**	1.489	3.264	2.773	1.141	1.129	1.110	1.1	1.125	**1.089**
CC	0.903	0.9	**0.940**	0.816	0.851	0.894	0.899	0.913	0.904	0.895	**0.928**

Note: Proteins, Interactions, Diameter, Degree, and Density indicate the number of proteins, the number of interactions, network diameter, average degree, and network density, respectively. ASP and CC are the average path length and clustering coefficient, respectively. Bold type indicates the minimum value on an average path length and the maximum value in the other topological properties of empirical and machine learning methods.

**Table 11:** Gene symbol and degree of the proteins that have the largest degree in every molecular complex of each network.

Strategies	Gene symbol	Degree
Union	RPL5, UBC	64

Intersection	IRAK1, IRAK2, and IRAK3	4

2-Vote	UCHL5	25

3-Vote	IKBKG	10

SVM	RPS8, RPS2, RPL5, RPL11, RPL18, RPS16, RPS6, RPL19, RPS13, RPL21, RPL6, RPL10A, UBC, RPS4X, RPL4, and RPS3	60

Naive Bayes	MED26, MED29	27

Random Tree	RPL5, UBC, and RPL4	39

J48	RPS2, RPL5, RPL11, RPS6, RPL19, RPL21, RPL6, RPL10A, UBC, RPS4X, RPL14, and RPL4	47

Logistic Regression	RPL5	65

Random Forest	RPL11, RPS6, RPL14, and RPL4	58

Bayesian Networks	RPL18, RPS16, RPS6, RPS4X, RPS8, RPS2, RPL5, RPL21, UBC, and RPL4	64
