# Supplementary material for: Integration Strategy Is a Key Step in Network-Based Analysis and Dramatically Affects Network Topological Properties and Inferring Outcomes
Source: Biomed Res Int. 2014 Aug 27;2014:296349. doi: 10.1155/2014/296349 (PMC4163410; doi:10.1155/2014/296349)
Supplement: Supplementary file 1 — OMIM descriptions of the top 10 disease genes that were detected by RWR on eleven integrated networks, seed genes for RWR algorithms were excluded. [file 296349.f1.docx]

**Supplementary Table S1 Description of the top 10 of all genes except the seeds of eleven integration networks in the** **detection of disease genes using RWR**

| **Gene symbol** | **MIM** | **Title (provided by OMIM)** |
| --- | --- | --- |
| ABL1 | 189980 | ABELSON MURINE LEUKEMIA VIRAL ONCOGENE HOMOLOG 1; ABL1 |
| APP | 104760 | AMYLOID BETA A4 PRECURSOR PROTEIN; APP |
| ARHGDIA | 601925 | RHO GDP-DISSOCIATION INHIBITOR ALPHA; ARHGDIA |
| ASF1A | 609189 | ANTI-SILENCING FUNCTION 1, S. CEREVISIAE, HOMOLOG OF, A; ASF1A |
| ASF1B | 609190 | ANTI-SILENCING FUNCTION 1, S. CEREVISIAE, HOMOLOG OF, B; ASF1B |
| CCDC90B |  |  |
| CDK2 | 116953 | CYCLIN-DEPENDENT KINASE 2; CDK2 |
| CTNNB1 | 116806 | CATENIN, BETA-1; CTNNB1 |
| DTNBP1 | 607145 | DYSTROBREVIN-BINDING PROTEIN 1; DTNBP1 |
| ELAVL1 | 603466 | EMBRYONIC LETHAL, ABNORMAL VISION, DROSOPHILA, HOMOLOG-LIKE 1; ELAVL1 |
| ERBB2 | 164870 | V-ERB-B2 AVIAN ERYTHROBLASTIC LEUKEMIA VIRAL ONCOGENE HOMOLOG 2; ERBB2 |
| EXO1 | 606063 | EXONUCLEASE 1, S. CEREVISIAE, HOMOLOG OF; EXO1 |
| FASLG | 134638 | TUMOR NECROSIS FACTOR LIGAND SUPERFAMILY, MEMBER 6; TNFSF6 |
| GRB2 | 108355 | TUMOR NECROSIS FACTOR LIGAND SUPERFAMILY, MEMBER 6; TNFSF6 |
| HNF4A | 600281 | GROWTH FACTOR RECEPTOR-BOUND PROTEIN 2; GRB2 |
| HRAS | 190020 | HEPATOCYTE NUCLEAR FACTOR 4-ALPHA; HNF4A |
| IMMT | 600378 | V-HA-RAS HARVEY RAT SARCOMA VIRAL ONCOGENE HOMOLOG; HRAS |
| MAX | 154950 | INNER MEMBRANE PROTEIN, MITOCHONDRIAL; IMMT |
| MLH1 | 120436 | MAX PROTEIN; MAX |
| MYC | 190080 | MutL, E. COLI, HOMOLOG OF, 1; MLH1 |
| PTPN6 | 176883 | V-MYC AVIAN MYELOCYTOMATOSIS VIRAL ONCOGENE HOMOLOG; MYC |
| RAD50 | 604040 | PROTEIN-TYROSINE PHOSPHATASE, NONRECEPTOR-TYPE, 6; PTPN6 |
| RB1 | 614041 | PROTEIN-TYROSINE PHOSPHATASE, NONRECEPTOR-TYPE, 6; PTPN6 |
| SDHA | 600857 | RAD50, S. CEREVISIAE, HOMOLOG OF; RAD50 |
| SHC1 | 600560 | RB1 GENE; RB1 |
| SMARCA4 | 603254 | SUCCINATE DEHYDROGENASE COMPLEX, SUBUNIT A, FLAVOPROTEIN; SDHA |
| TAF1 | 313650 | SHC TRANSFORMING PROTEIN; SHC1 |
| TNIK | 610005 | SWI/SNF-RELATED, MATRIX-ASSOCIATED, ACTIN-DEPENDENT REGULATOR OF CHROMATIN, |
| UBC | 191340 | TAF1 RNA POLYMERASE II, TATA BOX-BINDING PROTEIN-ASSOCIATED FACTOR, |
| VAV3 | 605541 | TRAF2- AND NCK-INTERACTING KINASE; TNIK |
| VIM | 193060 | UBIQUITIN C; UBC |
| XRCC6 | 152690 | VAV3 ONCOGENE; VAV3 |
| YWHAB | 601289 | VIMENTIN; VIM |
| YWHAE | 605066 | X-RAY REPAIR, COMPLEMENTING DEFECTIVE, IN CHINESE HAMSTER, 6; XRCC6 |
| YWHAZ | 601288 | TYROSINE 3-MONOOXYGENASE/TRYPTOPHAN 5-MONOOXYGENASE ACTIVATION PROTEIN, |
| ZHX1 | 604764 | TYROSINE 3-MONOOXYGENASE/TRYPTOPHAN 5-MONOOXYGENASE ACTIVATION PROTEIN, |
